# Emergence of crowding: The role of contrast and orientation salience

**DOI:** 10.1167/jov.21.11.20

**Published:** 2021-10-28

**Authors:** Robert J. Lee, Josephine Reuther, Ramakrishna Chakravarthi, Jasna Martinovic

**Affiliations:** 1 Cambridge Research Systems Limited, Kent, UK; 2 School of Psychology, University of Aberdeen, Aberdeen, Scotland, UK; 3 Department of Psychology, School of Philosophy, Psychology and Language Sciences, University of Edinburgh & School of Psychology, University of Aberdeen, Aberdeen, Scotland, UK

**Keywords:** color, luminance, crowding, orientation discrimination, contrast, S-cones

## Abstract

Crowding causes difficulties in judging attributes of an object surrounded by other objects. We investigated crowding for stimuli that isolated either S-cone or luminance mechanisms or combined them. By targeting different retinogeniculate mechanisms with contrast-matched stimuli, we aim to determine the earliest site at which crowding emerges. Discrimination was measured in an orientation judgment task where Gabor targets were presented parafoveally among flankers. In the first experiment, we assessed flanked and unflanked orientation discrimination thresholds for pure S-cone and achromatic stimuli and their combinations. In the second experiment, to capture individual differences, we measured unflanked detection and orientation sensitivity, along with performance under flanker interference for stimuli containing luminance only or combined with S-cone contrast. We confirmed that orientation sensitivity was lower for unflanked S-cone stimuli. When flanked, the pattern of results for S-cone stimuli was the same as for achromatic stimuli with comparable (i.e. low) contrast levels. We also found that flanker interference exhibited a genuine signature of crowding only when orientation discrimination threshold was reliably surpassed. Crowding, therefore, emerges at a stage that operates on signals representing task-relevant featural (here, orientation) information. Because luminance and S-cone mechanisms have very different spatial tuning properties, it is most parsimonious to conclude that crowding takes place at a neural processing stage after they have been combined.

## Introduction

It is difficult to judge the attributes of a visual object when other objects are nearby. This is particularly noticeable when objects are in the periphery of the visual field. There are at least two mechanisms that can cause this reduction in discrimination: (1) masking, whereby effective contrast is reduced due to another stimulus being placed in close proximity (for reviews, see Breitmeyer, 2008; Foley, 2019; Kahneman, 1968; Kouider & Dehaene, 2007), and (2) crowding, whereby the proximity of another stimulus reduces the discrimination of stimulus properties (for reviews, see Levi, 2008; Pelli & Tillman, 2008; Strasburger, 2020; Strasburger, Rentschler, & Jüttner, 2011; Whitney & Levi, 2011). Masking occurs earlier than crowding: it emerges at the stage of contrast processing while crowding emerges at the stage of discrimination of objects and their properties (e.g. Chung, Levi, & Legge, 2001; Lev & Polat, 2015; Levi, Hariharan, & Klein, 2002; Pelli, Palomares, & Majaj, 2004).

Both masking and crowding are tuned to specific chromoluminant channels (pattern pedestal masking: Chen, Foley, & Brainard, 2000a; Chen, Foley, & Brainard, 2000b; lateral masking: Huang, Mullen, & Hess, 2007; crowding: Kennedy & Whitaker, 2010). Kennedy and Whitaker (2010) suggested a locus for crowding effects beyond the level of chromatic opponent mechanisms. This is in line with the accepted view that crowding originates in cortical neurons (Afraz, Montaser-Kouhsari, Vaziri-Pashkam, & Moradi, 2003; Aghdaee, 2005; Flom, Heath, & Takahashi, 1963; He, Cavanagh, & Intriligator, 1996; for reviews, see Pelli, 2008; Pelli & Tillman, 2008). The majority of cortical neurons code for multiple features, including jointly processed color and luminance contrast in V1 and beyond (for reviews, see Gegenfurtner, 2003; Johnson & Mullen, 2016).

In humans, signals from three cone types that differ in their peak wavelength sensitivity (short – S, medium – M, and long – L) are recombined in pre-cortical, post-receptoral channels to separately derive luminance (L + M) and color information (L-M; S-[L + M]; Lee & Silveira, 2016). The chromatic S-(L + M) mechanism receives input from the sparser and more regularly distributed S-cone mosaic. This system has low temporal and spatial resolution (Hendry & Reid, 2000): its bandwidth overlaps with the two lowest luminance spatial frequency channels (Humanski & Wilson, 1993). Whereas S-(L + M) signals can still support mid and high-level vision processes on their own, they do so differently, and often less effectively, than luminance or L-M signals (contour integration: Beaudot & Mullen, 2001; Beaudot & Mullen, 2003; Mullen, Beaudot, & McIlhagga, 2000; symmetry perception: Martinovic, Jennings, Makin, Bertamini, & Angelescu, 2018; object classification: Jennings & Martinovic, 2014). On the other hand, Coates and Chung (2016) reported that crowding for stimuli defined solely by S-cone signals appears to be similar to crowding for luminance-defined low contrast stimuli. Thus, the signature of crowding remains the same irrespective of low-level input as long as luminance contrast is reduced to approximately match resolvability between the luminance and the low-acuity S-cone systems. That is, at least some spatial processes act similarly in the S-(L + M) channel as in luminance channels.

Nevertheless, in everyday viewing luminance and color signals tend to be colocalized (Johnson, Kingdom, & Baker, 2005). In these situations, one could assume that crowding would be mainly driven by the higher-acuity luminance signals, which would provide stronger cues for shape perception. However, the presence of chromatic contrast could enhance stimulus conspicuity, making it appear more salient. Recently, stimulus appearance has been proposed as a major contributor to crowding (Coates, Wagemans, & Sayim, 2017; Herzog, Sayim, Chicherov, & Manassi, 2015). Of course, conspicuity affects appearance quite differently from grouping. For example, if the same amount of S-cone driven color contrast was added to low contrast luminance-defined target and flankers, these stimuli would appear more salient due to enhanced stimulus appearance. Could this increase in salience across all objects (targets and flankers) modulate crowding through an increase in effective (i.e. perceived) contrast, even when the S-cone color contrast itself has no effect on the processing of other task-relevant spatial features (e.g. orientation)? There are two possible routes for such modulation. First, if a target is more conspicuous, it can be localized and processed more efficiently and thus more accurately when presented without flankers or with far-away flankers. Second, increased conspicuity could also modulate the salience of the outer flanker, whose suppressive influence (see Petrov & Meleshkevich, 2011a) may be more adversely impacted by low contrast. On the other hand, salience in terms of perceived contrast might matter much less than salience of the task-relevant feature (see Felisberti, Solomon, & Morgan, 2005).

To examine the role of conspicuity (perceived contrast) in shape discrimination processes and evaluate flanker interference under these conditions, we studied crowding with S-cone signals in isolation and in combination with luminance in two experiments. In this context, processing of S-cone signals is particularly interesting – large S-cone contrasts are needed for reliable object discrimination (Jennings & Martinovic, 2014; Jennings, Tsattalios, Chakravarthi, & Martinovic, 2016), although the appearance of the stimulus is altered at much lower contrast levels when S-cone signals are added to L-M or luminance signals. This allows color contrast to be used to alter appearance and potentially increase overall conspicuity (defined through perceived contrast) of relatively low-contrast luminance stimuli without altering the salience of task-relevant spatial features (e.g. orientation).

In the first experiment, we aimed to validate the findings of Coates and Chung (2016) about S-cone-driven crowding using a different stimulus setup and task and also to test if these findings extend to combined color and luminance stimuli. We expected to replicate the observation that S-cone crowding is similar to low contrast luminance crowding. If increased salience (i.e. increased conspicuity) through the addition of color also lead to increased crowding when luminance contrast is relatively low, we would also expect to see more crowding for stimuli that combined color and luminance. The second experiment was a follow-up, in which we aimed to capture basic determinants of performance (i.e. detection and orientation discrimination thresholds) and appearance (i.e. points of subjective equality for perceived contrast driven by luminance alone and a combination of luminance with color) on a larger sample of participants. This would allow us to assess how these factors relate to crowding for relatively low contrast stimuli. Again, if increases in perceived contrast due to addition of color lead to increased crowding, this should produce more crowding for combined color-luminance stimuli. More importantly, we intended to use an individual differences approach to evaluate the dependence of crowding on the ratio of stimulus contrast relative to contrast thresholds for feature detection and discrimination. This was motivated by our combined observation of (a) relatively flat crowding functions and (b) substantial individual variation in the amount of crowding in the first experiment, which relied on isoluminant and low-contrast luminance-defined stimuli. Previously, it has been reported that the classic signature of crowding is absent at low contrasts (6%; Simmers, Gray, McGraw, & Winn, 1999). Thus, we expected to see flatter crowding functions for those participants in whom the tested stimuli were closer to their detection and discrimination thresholds.

Coates and Chung (2016) could not distinguish if crowding was localized within retinogeniculate mechanisms or if it occurred after the information derived from these mechanisms was combined. Kennedy and Whitaker (2010) found reduced crowding between colors belonging to the opposite poles of cone-opponent channels (reddish/greenish and bluish/yellowish), placing the earliest possible site of crowding in cortical neurons that represent such half-wave rectified chromatic stimuli. Still, it is not clear whether crowding can emerge as early as the output stage of the cortical neurons receiving cone-opponent and cone-additive retinogeniculate inputs, or if it emerges at subsequent stages that represent various features (e.g. orientation, spatial frequency, or hue) on the basis of these signals. By assessing how individual differences in contrast detection, orientation discrimination and perceived contrast might relate to individual differences in crowding for low-contrast stimuli, we aim to contribute to the debate on the neural site(s) at which crowding emerges and thus provide valuable insights for models of crowding.

## Experiment 1: Crowding with S-cones

We assessed the attributes of crowding for S-cone isolating, low contrast luminance isolating and combined S-cone/luminance stimuli. In the experiment, we aimed to ensure comparability of performance across different mechanisms by setting the contrasts of targets and flankers to an equal multiple of target orientation discrimination thresholds. The main bottleneck was the need to display S-cone isolating stimuli at a contrast that would be as high as possible but still within monitor gamut. Mullen and colleagues (2000) presented their stimuli for 500 ms in their study of contour integration across S-(L + M), L-M, and achromatic mechanisms. We opted for the same stimulus duration, but collected eye movement data for three out of 11 experienced participants to verify that our observers strictly followed the instruction to fixate. Finally, to verify the visibility of our isoluminant and low luminance contrast stimuli, we also measured detection thresholds at target and flanker eccentricities.

We assessed flanker interference using displays with three oriented flankers and a 2IFC orientation discrimination task, in which the target Gabor element in the first interval was oriented between −5 degrees and +5 degrees (vertical = 0 degrees) and the second element was misoriented by a certain number of degrees from the first element; we controlled the level of misorientation using an adaptive staircase implemented through the Palamedes toolbox (Prins & Kingdom, 2009). Participants decided whether the second element was rotated left or right with respect to the first. The same task was used in McIlhagga and Mullen (1996) and Mullen et al. (2000). The task was performed without flankers and with flankers at several distances within and just outside the classically defined Bouma's window (i.e. half of stimulus eccentricity; although when overall contrast is reduced, Bouma's window has been reported to be extended, see Kooi, Toet, Tripathy, & Levi, 1994; Tripathy & Cavanagh, 2002). To reiterate our predictions, based on Coates and Chung (2016), crowding should be the same across S-cone isolating and luminance channels, as long as their contrasts are equated in relation to the feature discrimination threshold. There are two distinct predictions for the S-cone/luminance combination: (1) if only the feature-defining luminance channel matters, the pattern of crowding stays the same; and (2) if appearance matters, crowding might change due to the more salient appearance of target and distractors, with the increase in perceived contrast of suprathreshold stimuli leading to increased crowding.

### Methods

#### Participants

Eleven observers (6 men and 5 women; age ranging from 24 to 45 years) participated in the experiment. All had normal color vision, as evaluated by the Cambridge Colour Test (Regan, Reffin, & Mollon, 1994) and normal or corrected-to-normal visual acuity. Four of the observers are authors, whereas the rest were experienced psychophysical observers but naive to the purpose of the experiment. Observers gave written informed consent prior to taking part. The study was approved by the Psychology Ethics Committee of the University of Aberdeen and was in line with the Declaration of Helsinki.

#### Apparatus

For the eight participants who performed the experiment without eye tracking, stimuli were presented on a Mitsubishi DiamondPro 2070SB CRT display, driven by a CRS (Cambridge Research Systems Ltd., Kent, UK) ViSaGe system, giving 14-bit resolution per Red Green Blue (RGB) channel. Monitor output was calibrated prior to testing using a ColorCal2 (CRS, Kent, UK). The monitor was switched on at least 30 minutes before the start of the experiment. Observers viewed the display from a distance of 96 cm.

For the three participants who performed in the experiment with eye tracking, we used a different setup. This was necessitated by the fact that our eye tracker could not interface with a 32-bit Visage system. A Display^++^ (CRS) LCD display was used instead, driven by a ViSaGe (CRS) graphics card from a 64-bit PC. Maximal contrast in the tested color direction was slightly larger on the Display^++^, which has a somewhat broader color gamut. Eye movements were monitored using a Livetrack FM (CRS) video eye tracker for fixation monitoring. Participants viewed the display from a distance of 57 cm.

Participants performed the experiments with their head on a chinrest, and gave responses via a Cedrus-530 button-box (Cedrus, San Pedro, CA, USA). Measurements of monitor phosphors by a SpectroCAL (CRS) were used in combination with CIE 2006 cone fundamentals (CIE, 2006; Stockman & Sharpe, 2000) to ensure accurate color representation. CRS Toolbox and CRS Color Toolbox (Westland, Ripamonti, & Cheung, 2012) for Matlab (The Mathworks Inc., Natick, MA, USA) were used to run the experiment and to calculate RGB values to produce desired cone excitations.

#### Stimuli

All stimuli used in the experiment were comprised of Gabor patches (sinusoidal modulations of contrast along one spatial dimension, with a 2D Gaussian window), each subtending approximately 1 degree of visual angle. All contrasts were modulated around a background that was metameric to CIE illuminant D65 and had a luminance of 11.0 cd/m^2^. The sinusoidal component of these Gabor patches had a spatial frequency of 2 cycles per degree (cpd) and a phase that placed a peak in contrast at the center of the patch. The Gaussian window had a standard deviation of 0.18 degrees.

**Figure 1.**
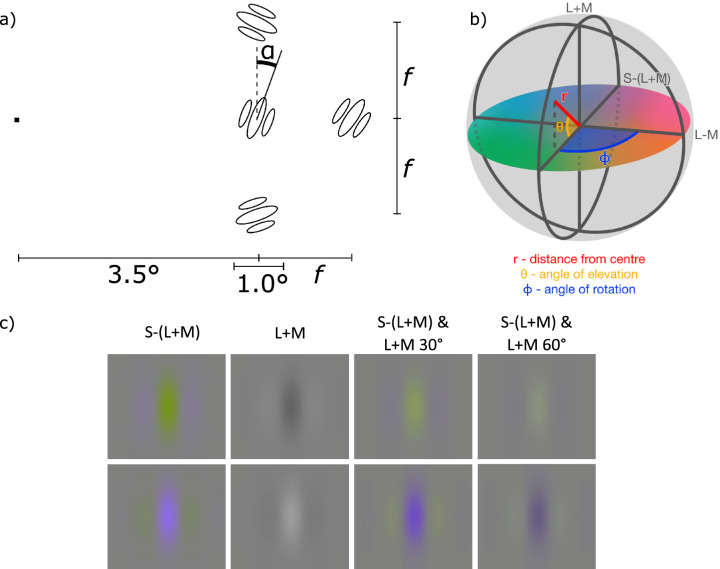
Stimulus Properties. (**a**) Spatial arrangement of stimuli: f indicates the distance from the center of the target Gabor to the center of each of the flankers (when present), α indicates the rotation of the target Gabor. Observers were instructed to fixate the dot (far left of this diagram) which was in the center of the display. The direction of the Gabors from the fixation dot, either left or right, was randomly chosen each trial, so in approximately 50% of trials, the arrangement was reversed horizontally. (**b**) The DKL (Derrington et al., 1984) space used throughout this paper. Its three main axes correspond to cardinal mechanisms: L + M, L-M, and S-(L + M). At the center of the space is the neutral gray, equivalent to the background level. The distance from the center is the radius (r), which corresponds to contrast. Hue is determined by the angle of rotation, while the angle of elevation determines the extent to which achromatic signals are present in the stimulus. If the angle of elevation is 0 degrees the stimulus is nominally isoluminant, and if it is +90 degrees or −90 degrees it contains only achromatic signals. (**c**) Examples of the Gabor patches in our stimuli showing the different chromatic contrast directions. From left to right, S-(L + M) isolating, luminance (L + M) isolating, S-(L + M), and L + M at 30 degrees luminance elevation and S-(L + M) and L + M at 60 degrees luminance elevation. The top and bottom rows show the opposite phases of the sinusoidal component, used in interleaved running-fit staircases. Note that within a staircase, target and flankers would have the same contrast on any single trial.

Observers fixated on a dot in the center of the display, and a Gabor patch designated as the target appeared 3.5 degrees away (or 5.25 degrees in some preliminary measurements), to either the left or right of the fixation dot. Three additional Gabor patches, designated as “flankers,” were placed one above and one below the target, and the third to the left of the target if the target was presented to the left of fixation or to the right if the target was presented to the right of fixation (the “outer” flanker). The distance between the target and flankers was different in different experimental sessions: 1.2 degrees, 1.4 degrees, 1.6 degrees, and 2.0 degrees (f in Figure 1a). Additionally, in another condition and for preliminary contrast measurements, no flankers were present. The orientation of each of the flanker Gabor patches was random in any stimulus. The orientation of the target Gabor was determined by the experimental procedure as described below. The contrast of the Gabor patches was defined along different chromatic directions in different experimental sessions. We define all our chromaticities in spherical Derrington-Krauskopf-Lennie (DKL; Derrington, Krauskopf, & Lennie, 1984) space (Figure 1b). In some sessions, the contrast was defined along the S-(L + M) axis (i.e. S-cone isolating), in some it was defined along the L + M (i.e. luminance isolating) axis, and in the remaining two sessions it was defined along chromaticity directions that combine S-cone and L + M contrast. These latter conditions combined S-cone contrast with L + M contrast defined by an angle of elevation above the isoluminant plane in DKL space (as in Jennings & Martinovic, 2014; Jennings et al., 2016). In the current experiment, we used angles of 30 degrees and 60 degrees. Examples of each of these contrasts are shown in Figure 1c. Contrast magnitudes (radius from the white-point origin in the DKL space) in the main experiment were set individually for each participant and for each chromatic direction based on their contrast thresholds for orientation discrimination, as described below. In some stimuli, the contrast was “positive,” placing an increase in, for example, S-(L + M) signal at the center of the Gabor so that it appeared to have a blue central stripe with yellow on either side, and, in some stimuli, the contrast was “negative,” giving the opposite color arrangement. In the stimuli consisting of both S-(L + M) and L + M contrast, the sinusoidal modulations were in-phase, meaning that increases in S-(L + M) coincided with increases in L + M so blue included more luminance than yellow. For some observers, we ran additional sessions with the two modulations in antiphase, so this relationship was reversed.

#### Procedure

**Figure 2.**
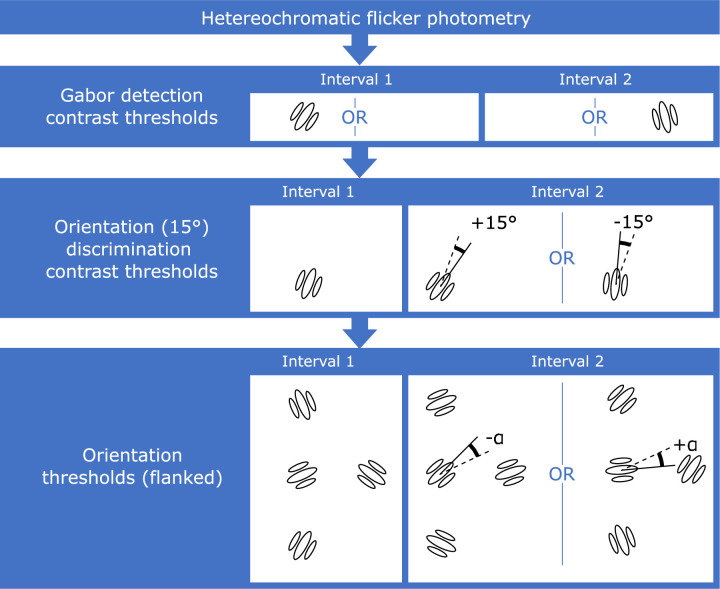
**Flowchart for the procedure followed in** Experiment 1**.** Note that images depict events in the first and second interval for each task. In cases where different events are possible, the word OR is displayed in between the alternatives. These were then displayed either on the left or right side of fixation. Heterochromatic flicker photometry was followed by detection contrast thresholds, in which one interval was blank and the other contained a Gabor patch. We then measured orientation discrimination contrast thresholds, with the first interval containing a near-vertical Gabor and the second interval containing a Gabor oriented to the left or right of the first interval Gabor. A single trial in the main flanker interference task (last stage in the flowchart) is also illustrated. Here, the task was the same as in orientation thresholds, but the dependent variable was the angle of orientation, rather than contrast, which was fixed to a suprathreshold value based on previous measurements.

Each observer first took part in several sessions designed to measure parameters to be used in the main experiment (Figure 2).

##### Heterochromatic flicker photometry

First, heterochromatic flicker photometry was used to correct for any residual luminance signals in stimuli designed to be S-(L + M) isolating. Observers viewed stimuli as described above, with a fixed contrast in the S-(L + M) direction chosen to be as large as possible within the monitor gamut and a randomly chosen small offset in luminance elevation angle. The contrast was reversed at 20 Hz. Observers adjusted the luminance elevation until the apparent flicker was minimized. Each observer performed 10 such adjustments, each time with a randomly chosen starting luminance elevation (range −4 degrees to 4 degrees), and presented at a randomly chosen side of fixation and flanker distance. Highest and lowest values were rejected as outliers and the mean of the remaining eight luminance elevation values was applied to all further S-(L + M) stimuli for that observer.

##### Contrast thresholds for detection

Second, the contrast required for observers to detect Gabors was measured. This was done to evaluate the visibility of the stimuli used in the main crowding experiment and compare them across the different chromatic and luminance channels. These thresholds were measured at the target location of 3.5 degrees from fixation and for an outer distractor location of 5.25 degrees from fixation. Crowding exhibits an inward/outward asymmetry, with the outer flanker being more disruptive to target processing (Petrov & Meleshkevich, 2011a; Petrov & Meleshkevich, 2011b). Therefore, it was important to verify that the outer flanker was sufficiently visible under the relatively low contrast levels used in this study.

Observers were instructed to decide which of two temporal intervals contained a Gabor. Each interval lasted 500 ms and was separated by 700 ms during which only the background and fixation dot were visible. The Gabor was randomly oriented each trial. An adaptive running-fit procedure (Palamedes Toolbox; Prins & Kingdom, 2009) controlled the contrast to find the point at which the observer gave approximately 81% correct responses, and was set to terminate after the trial-to-trial contrast change direction reversed 12 times. Two such adaptive staircases were run concurrently, with one having contrast reversed relative to the other (i.e. one had positive contrast and the other had negative contrast, as described above). On each trial, the staircase to be used was randomly selected. The running-fit procedures generate a Gaussian probability distribution that indicates the likelihood of threshold as a function of contrast after each trial. We took the mean of the distribution after 12 reversals as an estimate of threshold, and the standard deviation of the distribution as an estimate of error. We used the same method of estimating threshold on all the running-fit procedures used in this study, and, in most cases, the results of several estimates were averaged.

For three of the 11 participants, fixation was monitored for this and all of the following tasks. Trials could only be started when a fixation was detected at the location of the fixation mark. This triggered drift correction and the onset of the stimulus presentation. If during the trial, participant's gaze deviated by more than 2 degrees, the trial was discarded and the running fit not updated. A nine-point calibration sequence was used at the beginning of each block, and whenever participants could not fulfil the fixation criterion at the beginning of the trial. The calibration sequence also happened if five consecutive eye movements were recorded.

##### Contrast thresholds for orientation discrimination

Third, the amount of contrast required for detecting 15 degrees orientation differences in the target Gabor patches was measured, separately for each of the chromatic directions. Observers viewed target Gabor stimuli with no flankers in two temporal intervals. The timing parameters were the same as for the previous procedure for measuring contrast thresholds. In the first interval, the orientation of the target Gabor was randomly chosen so that the “stripes” were aligned between −5 degrees (anticlockwise) and 5 degrees (clockwise) relative to vertical (see α in Figure 1a; i.e. the direction of the sinusoidal contrast component was between −5 degrees and 5 degrees relative to horizontal). In the second interval, the target Gabor was randomly chosen to be either −15 degrees or +15 degrees relative to the first target Gabor. The observers’ task was to decide which direction this orientation difference was in. Adaptive running-fit staircases, with opposite contrasts, controlled the contrast as before. No differences were found between thresholds for the two reversed contrasts, so they were averaged. The resulting values, for each of the chromatic directions, were then used in the orientation discrimination threshold measurements.

##### Crowding: Orientation discrimination thresholds with and without flankers

The main objective of this experiment was to measure the smallest orientation difference between two successive target Gabors that could be discriminated in the presence of flankers. The procedure for measuring orientation discrimination thresholds was very similar to that just described for measuring contrast thresholds for discriminating a fixed orientation difference, and again the timing parameters were the same. The differences were that the orientation of the second target relative to the first was adjusted by the adaptive staircase while the contrast was fixed. This fixed contrast level was based on the value previously measured for orientation discrimination at that chromatic direction. The obtained contrasts were multiplied by the same factor to make all stimuli suprathreshold in terms of their orientation content. Our goal was to generate stimuli that would be equated in suprathreshold orientation salience for different color directions (S-cone, achromatic, or combined), enabling a fair comparison for orientation discrimination with and without flankers. The multiplication factor was 1.5 whenever possible. For some observers, such high contrasts in all chromatic directions were not within the display gamut so the highest achievable multiples were used. The multipliers used for these observers ranged between 1.20 and 1.48. We opted to keep these participants within the sample, as their data did not show a fundamentally different pattern. We reasoned that their inclusion was also justifiable on the ground that with multiples of about approximately 1.20, stimuli were sufficiently suprathreshold and equated across mechanisms (see Supplementary Material S1 for individual observer plots).

Again, two adaptive staircases with opposite contrast were run concurrently, and the observers’ task was to indicate the direction of the orientation difference. Different sessions were run for each chromatic direction and flanker distance.

### Results

#### Detection contrast thresholds

**Figure 3.**
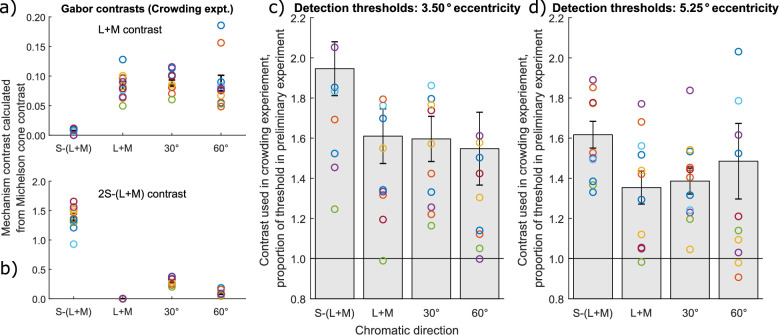
Stimulus contrasts in Experiment 1, depicting both contrasts used for obtaining orientation discrimination thresholds (left) and their ratios to detection thresholds at target and flanker locations. Contrasts in the L + M (**a**) and S-(L + M) (**b**) mechanisms used in the main crowding experiment, calculated from the cone fundamentals and measurements of the monitor primaries. Each colored symbol represents the same observer in all plots. Each observer has a slightly different contrast because slightly different multiples of their individual orientation threshold contrasts were used. The different stimuli are on the x-axis. Note that the L + M isolating stimulus has zero S-cone contrast, whereas the S-(L + M) targeting stimulus has approximately zero L + M contrast because of a minimal residual L + M signal produced by the monitor. Stimuli that include luminance signals have approximately the same L + M contrast values (top), but differ in the amount of S-(L + M) content, with L + M having none, 60 degrees elevation having very little and 30 degrees elevation having considerably more although much less than needed to drive task performance in the S-cone isolating stimulus. Contrast thresholds used in the crowding experiment, expressed as a proportion of the contrast required to detect the target Gabor at 3.50 degrees (**c**) and 5.25 degrees (**d**) eccentricities. Each circle symbol shows the threshold measured for a single observer, and the bar shows the mean over all observers. Error bars show ±1 standard error of the mean.

We compared the detection thresholds with the contrast used in the crowding task. The 5.25 degrees thresholds were lower than 3.5 degrees thresholds and in almost all instances (see Figure 3) remained below contrast levels used in the crowding experiment. That is, the stimuli used in the crowding task, targets, and flankers, were all suprathreshold.

#### Orientation discrimination contrast thresholds

We found no differences between the contrast thresholds measured with opposite phases (i.e. whether the center of the Gabor patch was bluish or yellowish, or light or dark). We also found no difference between thresholds for the 30 degrees and 60 degrees luminance elevation conditions with antiphase chromatic and luminance modulations (i.e. light bluish and dark yellowish versus dark bluish and light yellowish) when they were measured. Therefore, we averaged thresholds from sessions with corresponding chromatic direction and flanker distance.

#### Crowding: Orientation thresholds with flankers

**Figure 4.**
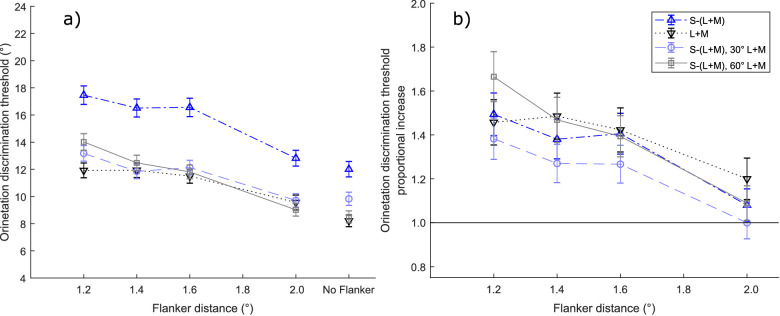
Orientation discrimination threshold results in Experiment 1. Mean orientation discrimination thresholds are depicted in (**a**), and the mean ratio of those thresholds to the thresholds when no flankers were present (threshold elevation) are depicted in (**b**). Separate lines indicate the four chromatic contrast directions of the stimuli, as indicated in the key. Error bars indicate 95% confidence intervals. In **b** the solid horizontal line indicates the ratio at which orientation discrimination thresholds with flankers would be the same as that without flankers.

Orientation thresholds averaged across all 11 observers are plotted in Figure 4a (see Supplementary Material S1 for individual observer plots). In all chromatic directions, orientation discrimination thresholds decreased with increasing flanker distances, and thresholds at 2 degrees spacing were similar to thresholds with no flankers. Thresholds for the S-(L + M) chromatic direction were higher at all flanker distances than for the chromatic directions containing a luminance component, but the latter conditions had similar thresholds.

Paired-samples *t*-tests were performed between thresholds measured with no flankers and with flankers at 2 degrees: (S-[L + M]: mean difference = 0.811 degrees, t(10) = 2.673, *p* = 0.023; L + M: mean difference: 1.352 degrees, t(10) = 3.109, *p* = 0.011; 30 degrees combined stimulus: mean difference = −0.116 degrees, t(10) = −0.339, *p* = 0.742; 60 degrees combined stimulus: mean difference = 0.518 degrees, t(10) = 0.556, *p* = 0.590). Based on this, there appears to be a slight elevation of orientation threshold for S-(L + M) and L + M mechanisms, but not for stimuli that combine signals from the two mechanisms. We take this to indicate that at a target-flanker spacing of 2 degrees, thresholds had more or less reached the unflanked level.

#### Crowding ratios (threshold elevation)

We took the ratio of the orientation thresholds in the presence of flankers to the thresholds without flankers, separately for each chromatic direction at each target-flanker spacing, for each observer. The means of these ratios across all observers are plotted in Figure 4b. The ratios decreased with distance, and at 2 degrees had reached roughly the same level as the thresholds with no flankers. However, interestingly, there was no overall effect of the chromatic direction on the threshold elevation ratio, as assessed with a linear mixed-model ANOVA (chromatic direction and flanker distance as fixed effects with flanker distance as a continuous variable (i.e. df = 1, and observer as a random grouping factor) on all the threshold ratios. There was a significant effect of flanker distance (F(1, 12.86) = 80.821, *p* < 0.001), but not of chromatic direction (F(3, 13.25) = 2.243, *p* = 0.131) or the interaction (F(3, 17.27) = 2.963, *p* = 0.061). The results of Bonferroni-corrected post hoc paired *t*-tests between flanker distances indicate that the major drop in performance occurs between 2 degrees and 1.6 degrees flankers (t(10) = 7.098, *p* < 0.001), but that subsequently performance remains fairly stable (1.6 degrees vs. 1.4 degrees: t(10) = 0.736, *p* = 1.00; 1.4 degrees vs. 1.2 degrees: t(10) = 2.497, *p* = 0.109).

### Interim discussion

In agreement with Coates and Chung (2016), we do not find any differences between signatures of crowding for S-cone isolating and low contrast luminance stimuli. Whereas Coates and Chung (2016) equated their tumbling E stimuli in terms of unflanked foveal acuity, we equated our Gabors in terms of parafoveal feature salience (i.e. orientation discrimination). The similar signature of performance – with different intercepts for S-cone stimuli and stimuli containing low contrast luminance, but with comparable influence of flankers – indicates that Coates and Chung's (2016) findings are robust enough to hold across different paradigms, as long as an attempt is made to establish a level of equivalence between chromatic and achromatic stimuli using a suitable metric. When the stimulus was defined using a combination of color and luminance, it was the luminance contrast that determined the orientation discrimination performance. This confirms the dominance of the high-acuity luminance signals for spatial vision tasks (e.g. Jennings & Martinovic, 2014). As luminance determines orientation discrimination, it also determines the extent of crowding, which is no different to what we observe for contrast-equated single channel stimuli. These findings also suggest that appearance, expressed in terms of perceived contrast, does not modulate crowding.

Mullen et al. (2000; see their Figure 7) tested uncrowded orientation performance for S-(L + M) stimuli at suprathreshold contrasts for a range of eccentricities (0 degrees−4 degrees). They observed considerably worse orientation discrimination for stimuli defined by S-(L + M) signals, but no deficit in curvature threshold or optimum path detection, which are important for sustaining contour integration. Based on this, Mullen and colleagues (2000) concluded that, although orientation discrimination is worse when defined by S-cone signals, contour integration across different retinogeniculate mechanisms is subserved by a common process. The same logic can be applied to crowding: there appears to be a general mechanism invariant to the type of contrast, ensuring that once thresholds are normalized to the uncrowded baseline (see Figure 4) there are no differences, whether the stimulus is defined by chromatic signals, achromatic signals, or any combination of the two.

On average, we do not observe a steep effect of crowding (see Figure 4). Flankers outside of Bouma's window (2 degrees) induce no or minimal interference with performance, as expected. The main costs arise when flankers enter just inside Bouma's window (1.6 degrees, with our target at 3.5 degrees) but there is only a small increase in crowding with further flanker proximity. However, the average plot conceals considerable individual variation (Supplementary Material S1): whereas, for some participants, crowding onsets at 1.6 degrees and remains relatively flat henceforth, for others, we observe much steeper flanker-interference functions inside Bouma's window. Steeper crowding is observed for three out of five participants for whom we used the 1.5 contrast multiplier and two out of six participants for whom we used a lower contrast multiplier. As effective contrasts differed between participants, and perceived contrasts were not empirically evaluated, further work was needed to understand why crowding with relatively low contrast stimuli may vary to such an extent.

As explained in the methods section, we used an eye tracker to monitor the gaze direction of three of the 11 participants, to ensure that they were maintaining fixation as instructed and not making eye movements toward the target Gabors. Although all participants were experienced and reported compliance with the instruction to fixate, stimulus duration of 500 ms did allow for target-directed saccades. Such saccadic behavior would have placed the stimuli closer to the fovea and reduced the effect of crowding. Despite this possibility, we observed similar patterns of orientation discrimination thresholds and threshold elevation ratios in these participants and the other experienced participants whose fixation was not monitored (see Supplementary Material S1), so we conclude that all participants were able to maintain fixation and that eye movements did not considerably influence the thresholds and crowding patterns observed in this experiment.

## Experiment 2: Crowding from luminance signals isolated or combined with color

In Experiment 1, we observed similar levels of crowding among low contrast luminance, S-cone isolating, and combined S-cone/luminance stimuli. However, as we did not quantify the increase in perceived contrast, it was not possible to know the extent to which conspicuity/salience increased due to the addition of color for different observers. There was also a considerable degree of individual variation in both crowded and uncrowded performance, similar to what has been previously reported (Greenwood, Szinte, Sayim, & Cavanagh, 2017; Kurzawski, Burchell, Thapa, Majaj, Winawer, & Pelli, 2021; Petrov & Meleshkevich, 2011a). However, although those studies report individual variability for high-contrast, suprathreshold stimuli, we observe variability at a range of contrasts that is much closer to feature-discrimination threshold. As mentioned earlier, crowding is nonexistent when contrasts are too low (Simmers et al., 1999). Therefore, the variability that we observe with stimuli that are just above contrast discrimination threshold may capture interesting information on the contrast-dependency of crowding.

To further evaluate the role of perceived contrast and to account for some of the observed variability, we turned to an individual differences approach. We recruited a larger participant sample (*n* = 24) and collected data on perceived contrast, detection, and discrimination thresholds for Gabor patch stimuli defined by luminance alone and luminance combined with some S-cone contrast. In the crowding task, we fixed the achromatic contrast to a relatively low value. We then measured performance for achromatic stimuli and stimuli that combined the same quantity of luminance contrast with an amount of S-cone defined color insufficient to sustain detection or discrimination on its own, but sufficient to alter the appearance of the Gabor patch from achromatic to chromatic (Jennings & Martinovic, 2014; Jennings et al., 2016). Thus, through using S-cone defined color, we can enhance conspicuity and perceived contrast of the stimulus without affecting detection and discrimination performance. This enables us to dissociate to a degree how detection/discrimination and appearance might affect crowding. To uncover the underlying factors that account for the combined variability of the investigated visual processes (detection, discrimination, perceived contrast, and crowding) and thus throw further light on the locus of crowding, we used factor analysis, a method capable of revealing visual mechanisms underlying performance (see Bosten, Goodbourn, Bargary, Verhallen, Lawrance-Owen, Hogg, & Mollon, 2017; Dobkins, Gunther, & Peterzell, 2000). We predicted we would identify two factors, with crowding being associated with both: one related to feature discrimination and the other related to appearance. In other words, we expected increased crowding to occur in those observers for whom stimuli were more conspicuous, both in terms of feature orientation and contrast.

### Methods

#### Participants

Twenty-four participants (7 men and 17 women; 16–41 years old, with mean age of 25 years) took part in the study. They were recruited from undergraduate researchers undertaking a semester-long practical in Dr. Martinovic's laboratory and by word-of-mouth among the experienced observers in the laboratory's participant pool (see Supplementary Material S2B for more detail). An additional participant was excluded because their detection thresholds surpassed the contrast levels to which the stimuli were fixed in the main experimental task. All participants had normal or corrected to normal visual acuity and normal color vision as evaluated by the City University color vision test (Fletcher, 1975). Participants gave written informed consent and either received class credit or reimbursement for taking part in the experiment. The study was approved by the Psychology Ethics Committee of the University of Aberdeen and was in line with the Declaration of Helsinki.

#### Stimuli and procedure

**Figure 5.**
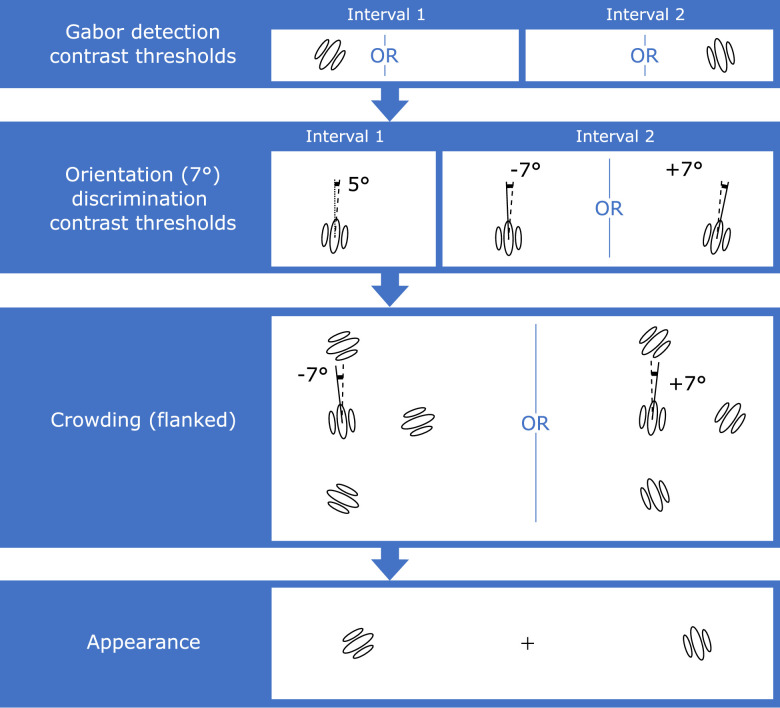
Flowchart for the procedure followed in Experiment 2. Contrast detection thresholds were followed by contrast orientation thresholds. Subsequently, participants performed the 1AFC flanker interference task. The final task was the measurement of point of subjective equality for perceived contrast.

The apparatus, stimulus and procedure (Figure 5) were very similar to those in Experiment 1. This section focuses on the differences between experiments, with the remaining parameters (e.g. size and spatial properties of the Gabors, and distance from fixation) remaining the same.

Stimuli were presented for 150 ms in order to eliminate the possibility of eye movements toward the stimulus (Salthouse & Ellis, 1980). Participants sat in a dark room at 93 cm in front of the monitor using a chin rest. They took part in four to five sessions, each lasting approximately 1 hour.

##### Detection and orientation contrast thresholds

Contrast thresholds for detection and orientation discrimination were measured using a 2IFC procedure. For the detection task, participants were presented with two intervals of which only one interval contained the stimulus. After viewing both intervals, participants had to respond by pressing one of two buttons on the button box. Gabor patches in the detection task were randomly oriented. For the orientation task, the first Gabor patch was oriented between −5 degrees (anticlockwise) and +5 degrees (clockwise) from vertical; the second Gabor patch was then oriented up to −7 degrees or +7 degrees relative to the first Gabor. Participants were required to judge which direction this orientation difference was in. For each task, two adaptive staircases controlling contrast were run simultaneously, implemented using the Palamedes toolbox for Matlab. The staircases terminated after 14 reversals and we used a Weibull function to obtain the threshold (81% correct).

##### Crowding

The crowding task was one-interval forced choice (1IFC) “yes/no” task. The target Gabor patch had an orientation of −7 degrees (anticlockwise or left) or +7 degrees (clockwise or right) relative to vertical; the flankers surrounding it were assigned random orientations. Participants had to indicate whether the target patch was tilted to the left or to the right using the button box. Stimulus presentation lasted for 150 ms and coincided with the fixation cross changing from black to white. There were nine target-flanker spacing conditions: an unflanked and eight spacing conditions (1 degrees, 1.1 degrees, 1.2 degrees, 1.3 degrees, 1.4 degrees, 1.5 degrees, 1.6 degrees, and 1.8 degrees). The crowding task consisted of 100 trials per flanker spacing. Hence, each participant completed a total of 900 trials for each type of contrast – luminance alone or combined with color, which were blocked. Within each of these, trials were randomly intermixed and divided into 10 blocks of 90 trials, with an additional 20 practice trials at the start. For the luminance only block, the L + M mechanism contrast was 0.15; the color/luminance stimulus additionally contained 0.31 S-(L + M) mechanism contrast. Contrasts were computed in the same way as in Jennings and Martinovic (2014): Michelson cone contrasts were calculated from cone excitations (Golz & MacLeod, 2003) and used to compute mechanism contrasts.

##### Appearance task

The appearance matching task relied on a spatial two alternative forced choice (2AFC) procedure (i.e. two stimuli were presented simultaneously on either side of the fixation cross). We used a 2AFC instead of 2IFC to allow for a simultaneous comparison of the two contrast levels, which we assumed would be more reliable than a comparison based on sequentially encoded representations and would eliminate any potential short-term memory contributions to the perceived contrast judgment. Participants were asked to select the stimulus they perceived to be more salient/“contrasty” by pressing the corresponding button on the button box. The standard was the “color + luminance” stimulus, which was fixed to the same contrast as used in the crowding task. Responses controlled a “one-up/one-down” staircase which adjusted the contrast of the luminance comparison and terminated after 20 reversals. We obtained the point of subjective equality (PSE) as the average of the last six reversals.

For the threshold and crowding tasks, the order of color/luminance conditions was counterbalanced across participants.

#### Data analysis

Detection and orientation contrast thresholds were used to assess the degree to which the stimuli presented in the crowding task were visible/discriminable – to do this, we expressed these contrasts in detection and orientation threshold units and then compared them using paired sample *t*-tests. Similarly, we expressed the PSE (obtained in the appearance task) in units of luminance contrast present in the color + luminance stimulus – this enables us to directly quantify the extent to which perceived contrast of the stimulus was enhanced by the addition of color. This test was done using a one-sample *t*-test against unity. Finally, d-primes were computed for each flanker distance in the main (crowding) task. Based on preliminary assessment of grand-mean performance (see results below), we applied linear fits to these data and used the slope as a measure of flanker interference.

It was our intention to enter the following variables into a factor analysis: (1) the ratio of stimulus contrast to detection contrast threshold, (2) the ratio of stimulus contrast to discrimination contrast threshold; (3) the PSE for the luminance-only and luminance + S-cone patches expressed in units of luminance contrast present in the stimulus; and (5) the slope of the linear fits, reflecting flanker interference. To evaluate if these variables had a suitable degree of shared variance, we used the Kaiser-Meyer-Olkin (KMO) measure of sampling adequacy (Dziuban & Shirkey, 1974). The KMO index shows whether the variables belong together psychometrically and is used to decide if the correlation matrix is suitable for factor analytical methods. It equaled 0.73 across all variables, with the following individual values: 0.38 PSE, 0.68 luminance detection ratio, 0.71 color + luminance detection ratio, 0.77 color + luminance orientation ratio, 0.80 luminance orientation ratio, 0.83 color + luminance flanker interference slope, and 0.85 luminance flanker interference slope. Values between 0.5 and 0.6 are acceptable, 0.6 to 0.69 mediocre, 0.70 to 0.79 middling, and values over 0.8 are considered to be excellent for factor analytic approaches (Cerny & Kaiser, 1977). As the value for PSE was unacceptable, we removed it from the analysis. This led to the increase of the KMO index to 0.79 overall. With six relatively precisely measured variables, our sample size should be adequate to obtain a reasonable solution (Mundfrom, Shaw, & Ke, 2005), which is corroborated by the high KMO value. We performed a principal component analysis (PCA) on the correlation matrix and kept all factors with eigenvalues over one, as is standard in factor analysis.

### Results

**Figure 6.**
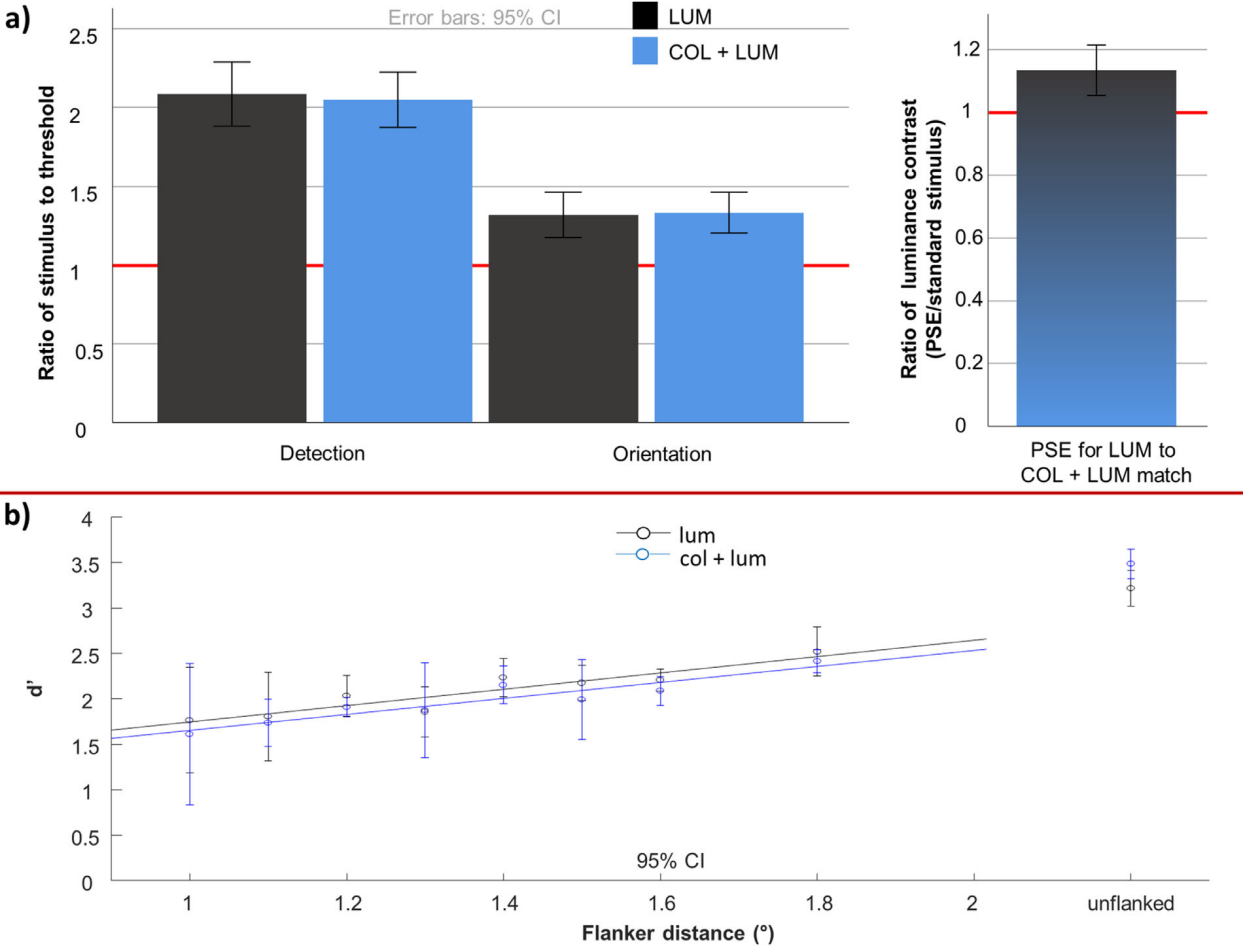
Detection and orientation thresholds, points of subjective equality for luminance and the combined (color + luminance; abbreviated as col + lum) stimulus and performance in the main crowding task. Top panel shows the stimulus contrast used in the main task expressed in multiples of detection and orientation thresholds (top left plot) and the PSE contrasts (top right plot). The stimulus is on average about twice above detection threshold and about 1.3 times above orientation threshold. The point of subjective equality is at about 1.1 times the luminance contrast contained in the combined (color + luminance) stimulus, which was the standard stimulus in the PSE task and equaled the color + luminance stimulus used in the crowding task. The lower panel shows performance in the crowding task, expressed in terms of discriminability (d’). Linear regression on the performance data, separately for luminance and color + luminance stimuli and not including the unflanked values, are shown with solid lines. Error bars indicate between-subject 95% confidence intervals. Performance appears to be highly similar for both luminance and the combined (color + luminance) stimulus. The classical Bouma's window (50% of eccentricity) would be at 1.75 degrees. Performance for the flanker just outside Bouma's window (1.8 degrees) is drastically below unflanked performance, indicating a large degree of flanker interference even at this relatively large target-flanker spacing.

Detection, orientation, and PSE contrast thresholds are presented in Table 1 and visualized in Figure 6, expressed as a ratio of stimulus contrast in the main crowding task (i.e. ratio of the stimulus contrast in crowding to these thresholds). There was no difference between the detectability of the stimulus (M_lum_ = 2.09, SD_lum_ = 0.48; M_col+lum_ = 2.05, and SD_coll+um_ = 0.42; t(23) = 0.72, *p* = 0.48) or discriminability of its orientation (M_lum_ = 1.32,SD_lum_ = 0.34, M_col+lum_ = 1.33, and SD_col+lum_ = 0.31; t(23) = −0.19, *p* = 0.85) between luminance alone and color + luminance. Orientation threshold ratios were lower than detection threshold ratios for both luminance (t(23) = 8.13, *p* < 0.001) and color + luminance (t(23) = 11.26, *p* < 0.001). Thus, stimuli were, on average, approximately twice above detection threshold, but only about 1.3 times above the orientation discrimination threshold. Finally, to achieve subjective equality with a color + luminance patch of a fixed contrast (as used in the main crowding task), participants slightly increased the amount of luminance contrast to a value that was higher than the luminance contrast contained within the color + luminance stimulus (M = 1.135, SD = 0.19, t(23) = 3.48, *p* = 0.002). These findings validate our approach in that they show the following: (1) detection and orientation thresholds depend only on the amount of luminance contrast rather than the added S-cone defined color contrast; (2) despite that, addition of color generally increases the salience of the stimulus, although only by approximately 14% on average for the small amount of chromatic, S-(L + M) contrast used in this study.

**Table 1.** Mean contrast thresholds (standard deviation) for detection and orientation discrimination in Experiment 2. The stimulus in the main crowding task had L + M contrast of 0.15 with an additional 0.31 S-(L+M) contrast in the color + luminance stimulus.

		Detection	Orientation	PSE
Luminance	L + M contrast	0.077 (0.018)	0.125 (0.037)	0.17 (0.030)
Color + luminance	L + M contrast	0.067 (0.046)	0.118 (0.031)	
	S-(L + M) contrast	0.159 (0.032)	0.248 (0.064)	

#### Crowding task

The data from the main, crowding task is shown in the lower panel of Figure 6. The figure shows that performance follows largely a linear trend, never fully reaching uncrowded performance. This is probably due to the relatively low contrast of the stimuli (see detection and orientation threshold ratios above). Paired *t*-tests between unflanked d's (d-primes) and d's at a target-flanker distance of 1.8 degrees (the furthest distance tested) confirm that flankers continue to impact performance at this distance, both for luminance alone (t(23) = 3.04, *p* = 0.006) and for color + luminance (t(23) = 3.40, *p* = 0.002) conditions. Meanwhile, there were no differences in unflanked performance between luminance alone and color + luminance, indicating that basic difficulty was matched across the two types of stimuli (t(23) = 1.11, *p* = 0.28). Because the data appeared to follow a linear trend, without ever reaching an uncrowded equivalent asymptote, we used slopes derived from linear fits of d’ as a function of target-flanker distance to evaluate the amount of interference from flankers. We found no difference between luminance alone and color + luminance in the amount of flanker interference (t(23) = 0.10, *p* = 0.92), finding the data between participants to again be quite variable (M_lum_ = 0.90, SD_lum_ = 0.99; M_col+lum_ = 0.88, and SD_col+lum_ = 0.78).

##### Factor analysis

Factor analysis yielded a single factor with an Eigenvalue over one, meaning that a single factor was sufficient in capturing the shared variability between our variables. This factor had an Eigenvalue of 3.4 and explained 57% of the variance. Correlations of the factor with individual variables were as follows: 0.82 luminance detection ratio, 0.88 color + luminance detection ratio, 0.68 luminance orientation ratio, 0.82 color + luminance orientation ratio, 0.65 luminance flanker interference slope, and 0.63 color + luminance flanker interference slope. Correlations above 0.40 are taken to be sufficiently high to be informative. The fact that all variables load positively on a single factor, which could thus be labeled “achromatic contrast driven performance,” means that for those participants for whom the stimuli were more above threshold, in terms of their visibility and orientation sensitivity, a more typical interference from flankers was observed, as indicated by accompanying steeper slopes in the crowding task.

**Figure 7.**
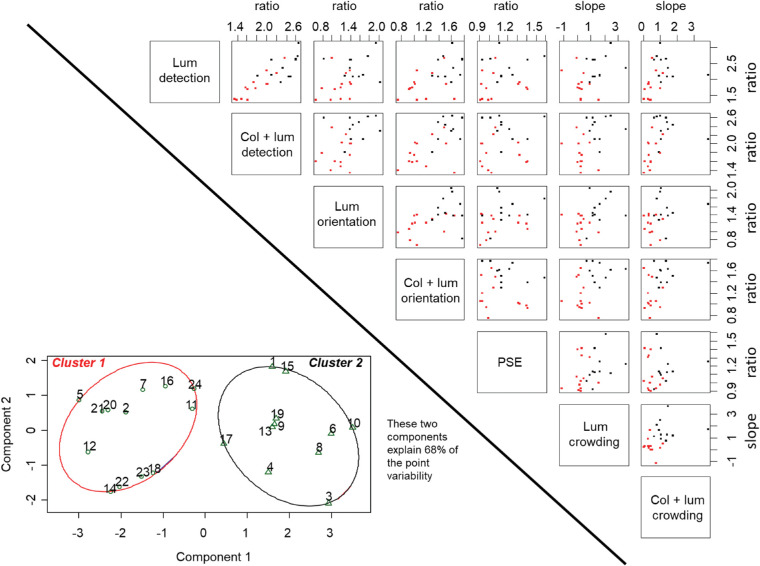
**Individual variability across thresholds, PSE** **,** **and flanker interference.** On the right, scatterplots that depict relations between task performance. On the lower left, two clusters that emerged from the K-means analysis are shown. In the scatterplots, participants falling into the first cluster are shown in red and participants falling into the second cluster are shown in black. It can be seen that the first cluster is in general performing worse on a wide range of these tasks, with those data points accumulating mainly in the lower left of each scatterplot. In the clustering diagram, each participant is depicted by their number.

Due to its insufficient correlation with the other variables, we had to leave perceived contrast out of the factor analysis. To cast further light on individual variability, we performed post hoc K-means clustering, in which we also included the PSEs. We wanted to assess if we could meaningfully divide participants into groups according to their patterns of individual differences. We included detection threshold ratios, orientation threshold ratios, flanker interference slopes and the PSE in this analysis. Clustering converged in two iterations, providing two groups, with a distance of 1.92 units between their centers. The first group had 13 members and the second group had 11 members. Figure 7 depicts the clusters using the visualization approach recommended by Pison, Struyf, and Rousseeuw (1999; implemented in R by Maechler, 2019): PCA is applied to the data and a bivariate plot of the participants is displayed relative to the first two principal components, outlining the clusters with ellipses.

**Figure 8.**
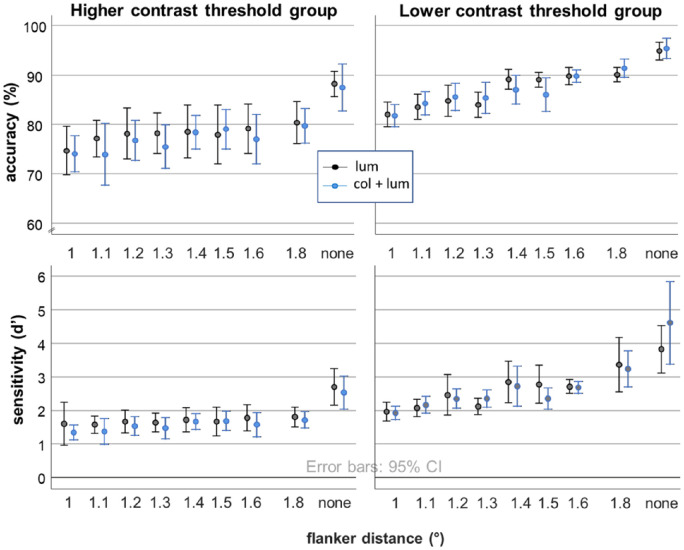
The effect of flanker interference for the two clusters – the higher threshold (i.e. lower sensitivity) group and the lower threshold (i.e. higher sensitivity) group. Accuracies are depicted in the top row, with sensitivities (d's) in the bottom row.

The groups differed significantly along all experimental variables apart from the PSE, as depicted in Table 2 below. This could have been expected, as the factor analysis already revealed correlations between all variables apart from the PSEs. Crowding in these two groups is depicted in Figure 8. The first group does not experience typical crowding, with flanker interference having a substantial but relatively stable impact on performance irrespective of distance. Their contrast threshold ratios are closer to one, particularly for orientation discrimination. The second group experiences flanker interference that increases with stimulus proximity and thus follows the expected signature for crowding (again, depicted in Figure 8). For this group, stimuli in the crowding task were approximately 1.5 times above orientation and approximately 2.4 times above detection threshold. From Figure 8, it is evident that performance between the two groups differs in several important aspects. First, the cluster with higher contrast thresholds is about 10 percentage points poorer, or almost half in terms of sensitivity, in the absence of any flankers. Second, flankers seem to produce a general cost in performance in this group, reducing performance by roughly the same amount irrespective of distance. Third, in the cluster with lower contrast thresholds, on the other hand, d's are almost matched for 1.8 degrees flankers and no flankers, with a more graded flanker interference, as expected from typical crowding interactions. The drop in performance for the 1.8 degrees flanker in Figure 6 is thus likely to be driven by the low-performing, high threshold group of participants, identified through our clustering approach.

**Table 2.** Results of the clustering analysis, depicting the center of each cluster and a one-way ANOVA evaluation on whether these differences are significant or not.

Variable	Cluster 1 center	Cluster 2 center	One-way ANOVA
Luminance detection ratio	1.81	2.41	F(1,22) = 14.17, *p* = 0.001
Color + luminance detection ratio	1.78	2.37	F(1,22) = 23.17, *p* < 0.001
Luminance orientation ratio	1.15	1.52	F(1,22) = 9.78, *p* = 0.005
Color + luminance orientation ratio	1.14	1.56	F(1,22) = 20.59, *p* < 0.001
PSE ratio	1.09	1.18	F(1,22) = 1.29, *p* = 0.27
Luminance crowding slope	0.28	1.64	F(1,22) = 21.33, *p* < 0.001
Color + luminance crowding slope	0.46	1.37	F(1,22) = 12.25, *p* = 0.002

From Figure 7, it can be seen that the majority of our participants judge the PSE to be within 10% of luminance contrast actually contained within the color + luminance stimulus (i.e. ratio of 0.9–1.1), with only eight participants adding 20% or more of luminance contrast. However, even these eight participants are split evenly into the two clusters, confirming that perceived contrast did not relate to the other variables that characterized low and mid-level performance. Further detail on the clustering approach, including a comparison of the two clusters to groups created by median splits along each variable, is given in Supplementary Material S2.

### Interim discussion

We observe a linear increase in performance with increasing distance between the flankers and the target, with evidence of interference even at the furthest flanker distance tested (at 1.8 degrees). Both neurophysiology and psychophysics indicate that receptive field size expands at low contrast (Mareschal & Shapley, 2004) and the same is known to be the case with integration field sizes (Kooi et al., 1994). Consistent with previous reports on the absence of typical crowding-like performance patterns at low contrast (Coates, Chin, & Chung, 2013; Simmers et al., 1999), we observe that the group of observers who were close to threshold in terms of discriminating the task-relevant orientation feature also showed flanker interference slopes close to zero. The ability to extract orientation information seems to be more important than more basic visibility, which remains suprathreshold, at approximately 1.5 times detection threshold, even in this group. The identified feature discrimination bottleneck is consistent with findings on detection and discrimination of oriented elements – whereas detection is driven by the most excited orientation-tuned neural templates, discrimination is determined by comparison of signals from differently oriented templates (e.g. Regan & Beverley, 1985, see also Solomon, 2002).

In our sample, about half belonged to the high-performing, low contrast threshold group and the other half to the low-performing, high contrast threshold group. The former displayed a clear signature of crowding whereas the latter did not. This might be taken to imply that participants for any crowding experiment must be prescreened to ensure that they can reliably display a signature of crowding, or that a substantial proportion of the population does not demonstrate “typical” crowding. However, that is not the case. What our results show is that, for any given participant, if the stimuli being used for examining crowding are at or close to that participant's discrimination threshold, then typical crowding will not be observed. However, for the same participant, if the stimuli were suprathreshold, the usual pattern of crowding results will be observed. Almost all crowding studies use suprathreshold stimuli and hence crowding should be reliably observed in all participants (e.g. Kurzawski et al. 2021). Even with low-contrast stimuli, if the contrast is tailored to exceed each individual participant's discrimination threshold (say by setting it at a specific multiple of unflanked discrimination contrast threshold), crowding should be observed. However, if one were to use stimuli with a fixed and low contrast across all participants (i.e. not tailored to each participant), it is possible that some participants will fail to demonstrate crowding, and the results might be not attributable to pure crowding processes. Future studies should define the limits of such behavior experimentally, by evaluating crowding at different multiples of discrimination threshold contrast, including values at or slightly below threshold.

We do not find any effect of appearance, operationalized in terms of differences in perceived contrast between luminance and color/luminance stimuli. This was contrary to our prediction that appearance should play a role, but is consistent with previous reports that stimulus salience has (at best) a modest effect on crowding (Felisberti et al., 2005). It could be argued that the magnitude of appearance change was insufficient to cause any effect. However, our clustering analysis shows that even the eight observers who show more substantial differences in appearance (adding >20% contrast to the luminance stimulus to match its appearance with the color + luminance stimulus) are split evenly between the low performing group without classical crowding and the high performing group with a more typical crowding signature. Perhaps an effect of conspicuity might have emerged if stimuli were not blocked by color. Interleaved with less conspicuous low-contrast luminance stimuli, combined color + luminance stimuli might have benefited more from higher perceived contrast. However, it is highly likely that appearance in terms of spatial grouping, as targeted by Manassi, Herzog, and colleagues (e.g. Herzog et al., 2015; Manassi, Sayim, & Herzog, 2013) is more relevant than appearance simply in terms of perceived contrast. Appearance understood in these former terms would allow flankers to be segmented into different layers when they do not group with the target, “freeing” the target from their contextual modulation (Doerig, Bornet, Rosenholtz, Francis, Clarke, & Herzog, 2019; Doerig, Schmittwilken, Sayim, Manassi, & Herzog, 2020; Francis, Manassi, & Herzog, 2017), which is very different than appearance as defined by perceived contrast.

At this stage, it is also relevant to consider recent findings that crowding for hue emerges independently from crowding for orientation, spatial frequency and motion (Greenwood & Parsons, 2020; Yashar, Wu, Chen, & Carrasco, 2019). This may be taken to imply that the pooling mechanisms for spatial information and color are fully separable. However, visual representation of color is not a process with a singular outcome. In other words, hue is only one dimension extracted by the color processing neural mechanisms. Shapiro's model (2008) demonstrates that color appearance and color contrast are dissociable, feeding into distinct neural processing modules. By adding S-cone contrast to a luminance Gabor, we alter surface appearance but do not alter the contrast from which spatial information is extracted. Within a single crowding display, our targets and distractors have the same appearance – either greyscale, chromatic, or a combination of the two (e.g. dark bluish to light yellowish). There is evidence that the degree of grouping by hue or by spatial stimulus properties affects crowding (or uncrowding). Previous work indicates that spatial grouping between target and flankers (Chakravarthi & Pelli, 2011; Livne & Sagi, 2007; Saarela et al., 2009; Saarela et al., 2010) strongly modulates crowding. Similarly, color imposes strong grouping cues that enact an early effect on global motion integration (Martinovic, Meyer, Muller, & Wuerger, 2009; Wuerger, Ruppertsberg, Malek, Bertamini, & Martinovic, 2011) and crowding (Põder, 2006; Manassi, Sayim, & Herzog, 2012). It would be interesting to see how grouping by spatial position and by color would interact in a crowding setting.

## General discussion

Through using S-cone isolating, luminance isolating, and combined S-cone and luminance stimuli, we can cast further light on the constraints for the emergence of crowding, which is informative of its neural locus. The low-level bottleneck for the emergence of crowding seems to be the ability to reliably discriminate stimulus orientation, as reflected by orientation discrimination contrast thresholds in our second experiment. Once orientation of a Gabor can be reliably discriminated, oriented flankers begin to enact a graded influence on performance, with less interference at further distances – a signature of crowding. In participants who exhibit a relatively strong flanker interference that is invariant to distance from target, task-relevant orientation information is represented less robustly.

It is useful to consider that the same opponent processes that underlie orientation discrimination also give rise to two of its very important attributes: (1) the precision of orientation tuning, which is several orders of magnitude higher than the precision within basic orientation-tuned detectors and (2) invariance to contrast, once the stimulus is sufficiently above contrast threshold (approximately 3 times and over; Regan & Beverley, 1985). Strasburger and colleagues have previously argued for the necessity of models to consider contrast encoding as the first stage that provides constraints to the more often considered “feature combination” stage, with crowding only emerging at “surplus contrast” (Chung et al., 2001; Levi et al., 2002; Pelli et al., 2004; Strasburger et al., 2011). In our study, we specify some of the important attributes of processing at the interface between the first, contrast-processing and the second, feature combination stage.

Orientation tuning in the S-cone pathway is less robust when compared to the achromatic pathway, leading to a basic cost in discrimination performance, but similar levels of flanker interference when compared to the achromatic pathway. Based on these combined findings – (1) flanker interference exhibiting a clear signature of crowding only when orientation discrimination threshold is reliably surpassed; (2) S-cone orientation discrimination being less robust, but crowding exhibiting the same signature for S-cone and achromatic signals at comparable levels of contrast – it is more parsimonious to conclude that crowding emerges after the extraction of featural (here, orientation) information, rather than occurring as an outcome of processing within each of the three different retinogeniculate mechanisms independently. If crowding had to emerge independently within each retinogeniculate channel, this would warrant grouping modulations (as demonstrated by the work of Herzog and colleagues; for models, see Doerig et al., 2019; Francis et al., 2017) to also be able to emerge within each channel independently, or else have similar feedback projections to each channel. Such assumptions are not as parsimonious. Our findings of crowding only emerging after reliable orientation discrimination is established are consistent with models that posit a contribution of both simple and complex cells to mid-level vision (Wilkinson, Wilson, & Ellemberg, 1997) and broadly consistent with a recent, mechanistic general model of human pattern perception that distinguishes between the operation of visual detectors under low and high-contrast constraints (Neri, 2018).

## Supplementary Material

Supplement 1

Supplement 2
