# Supplementary material for: Emergence of crowding: The role of contrast and orientation salience
Source: J Vis. 2021 Oct 28;21(11):20. doi: 10.1167/jov.21.11.20 (PMC8556554; doi:10.1167/jov.21.11.20)
Supplement: Supplement 1 [file jovi-21-11-20_s001.docx]

**Supplementary Material 2**

**Additional Between-subject Analyses for Experiment 2**

1. **Median Splits**

To further evaluate factors influencing the allocation of participants to clusters, we performed median splits for detection thresholds, orientation thresholds and flanker interference slopes. We then calculated Cramer’s V as a measure of the strength of association between low and high performing groups (as reflected by being below or above median) between each individual variable, on one hand, and the two clusters obtained through k-means clustering, on the other hand.

Cramer’s V quantifies the association of two variables as a percentage of their maximum possible variation. We surmised that if the clustering indeed separated participants efficiently into groups on the basis of all these variables, clusters should, at least to some degree, correspond to median splits, indicating a degree of reliability in the approach.

As can be seen from the table and the graph below, median splits and clustering share 59-75% of variation, with only 3-5 out of 24 participants not overlapping between the median split-created and clustering-created groups. Note that, as one cluster has 13 and the other has 11 participants, at least 1 participant would by default not correspond with an equal split of participants into two groups. Therefore, generated clusters are coextensive with low and high performing groups on each individual variable.

Supplementary Table 1 – Cramer’s V for median splits and clustering

|  | Cramer’s V, p value (N=24) |
| --- | --- |
| Luminance detection threshold | 0.585, p = .004 |
| Colour+Luminance detection threshold | 0.753, p<.001 |
| Luminance orientation threshold | 0.585, p = .004 |
| Colour+Luminance orientation threshold | 0.664, p = .001 |
| Luminance flanker interference slope | 0.753, p<.001 |
| Colour+Luminance interference slope | 0.753, p<.001 |

Supplementary Figure 1 – Comparison of median splits (below and above median) and clustering (1 – low performing group, 2 – high performing group).


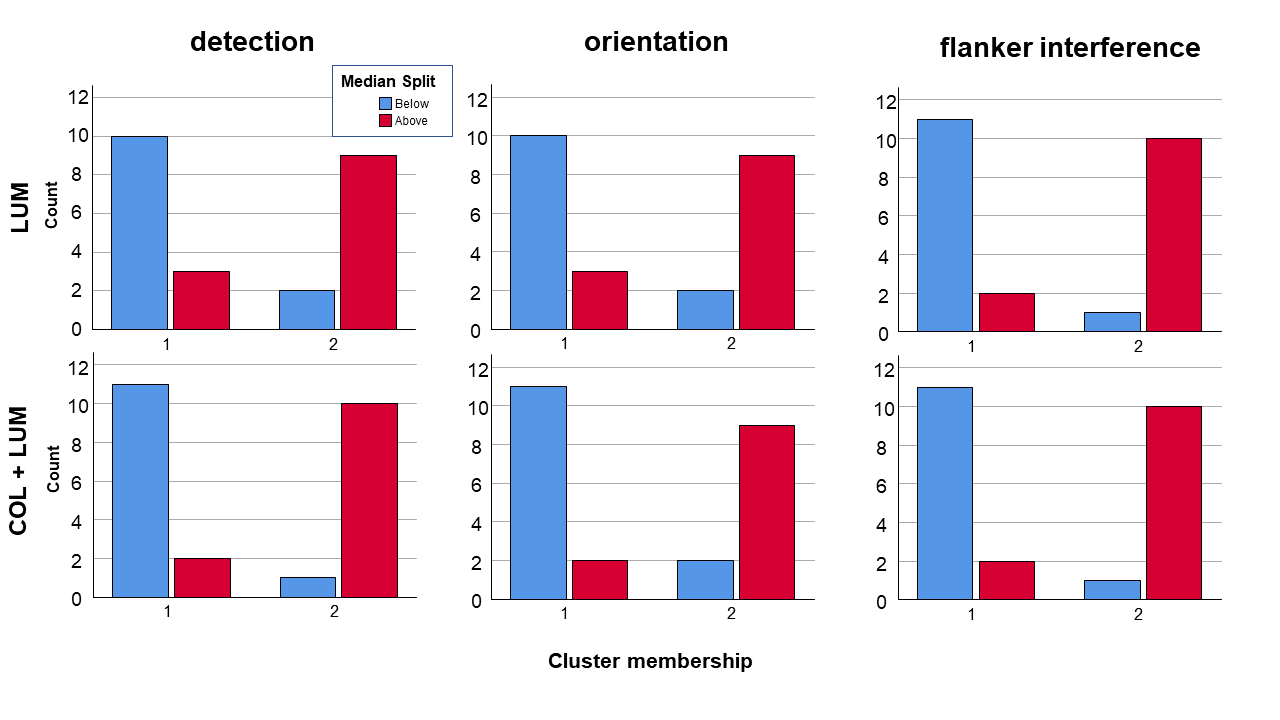


1. **Observer experience**

The participants in this experiment were a mix that included 12 experienced observers that have taken part in many psychophysical experiments and 12 undergraduate observers who were less experienced. These students were undertaking a semester-long research project in Dr Martinovic’s lab. While these observers were motivated and had practice with the tasks prior to participating, they still differed in their level of experience.

To evaluate statistically if the factor of experience overlaps with the two groups that came out of the clustering analysis, we categorised the 12 participants taking part in research practicals as ‘less experienced’ and 12 participants who had previous experience in taking part in psychophysical experiments as ‘more experienced’. We performed a chi-square analysis to evaluate whether these groups overlapped significantly with our two clusters, but there was no evidence for this (χ2(1)=-0.168, p=.682; see plot below).


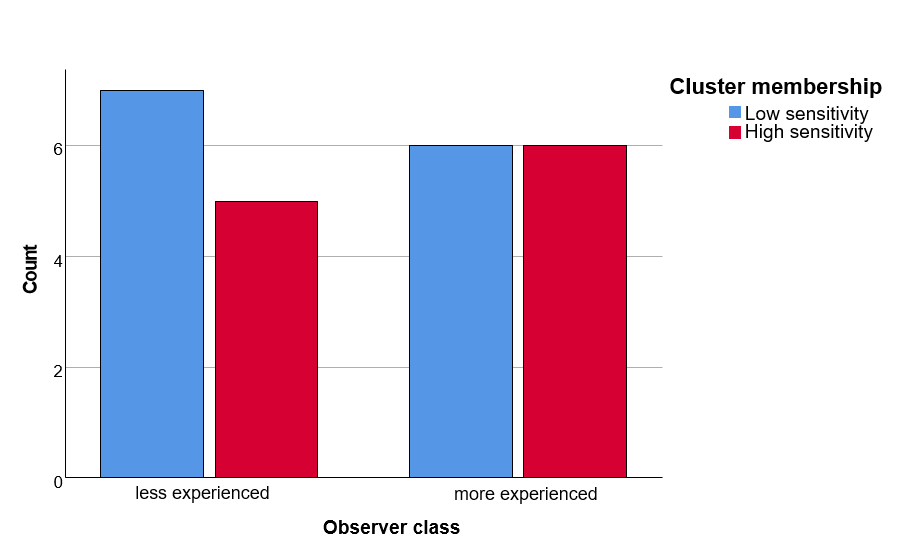


We also evaluated if the less experienced group had higher thresholds. However, this was not the case (LUM detection contrast threshold ratio: t(22)=-0.673, p=.508; LUM contrast orientation threshold ratio: t(22)=-0.662, p=.515).
