# Supplementary material for: Emergence of crowding: The role of contrast and orientation salience
Source: J Vis. 2021 Oct 28;21(11):20. doi: 10.1167/jov.21.11.20 (PMC8556554; doi:10.1167/jov.21.11.20)

## Supplementary Material 1

## Mean orientation discrimination thresholds for each individual observers in Experiment 1. Formatting is the same as the left panel of Figure 3 in the main text. Separate lines indicate the four chromatic contrast directions of the stimuli, as indicated in the key. Error bars indicate the standard deviations of the probability distribution that estimates the threshold, generated by the running fit procedure (averaged over the two staircases).

Key to symbols:


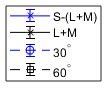


## Observers with threshold contrast multiplier 1.5

Observer: 1 (no eye tracking)


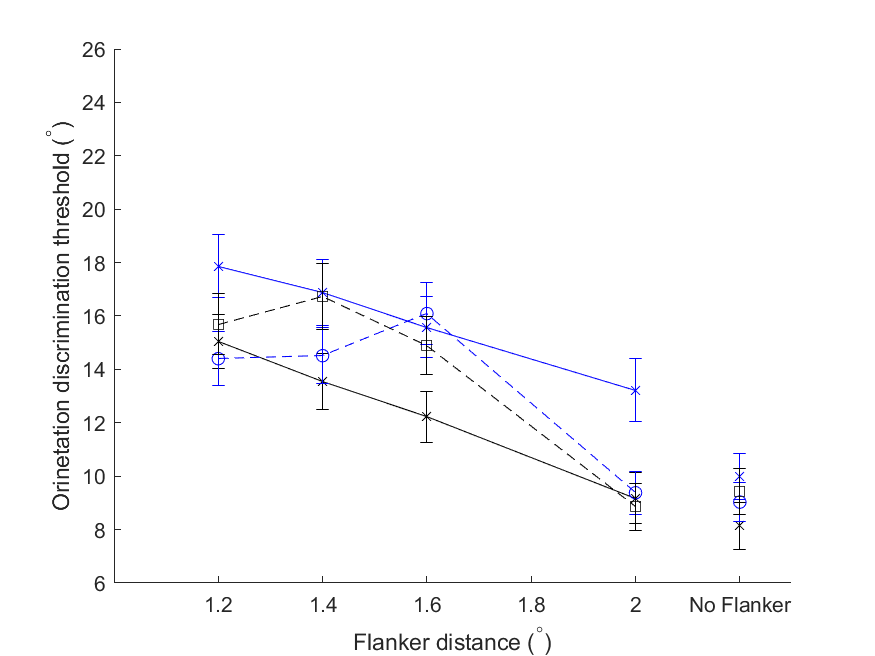


Observer: 2 (no eye tracking)


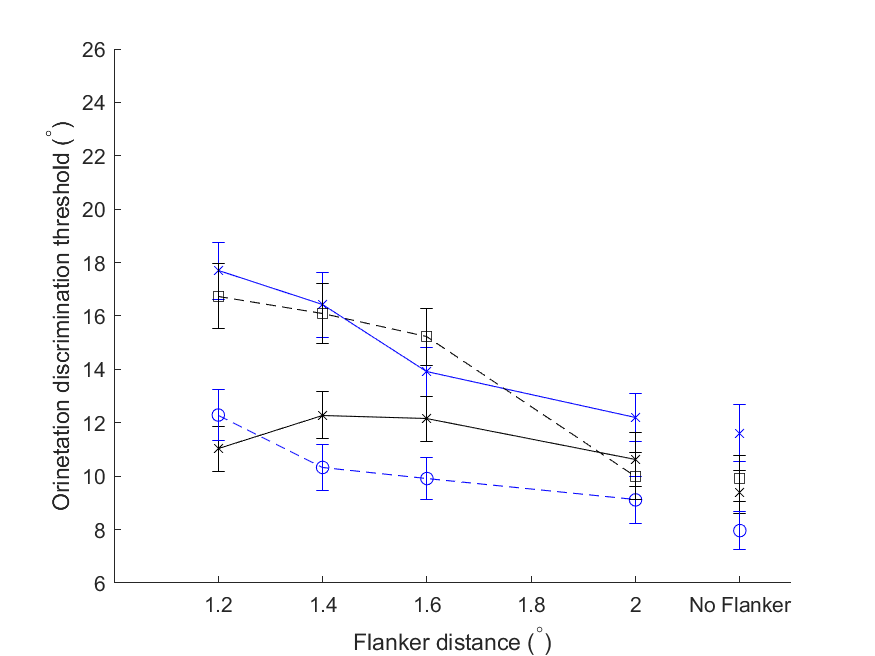


Observer: 3 (no eye tracking)


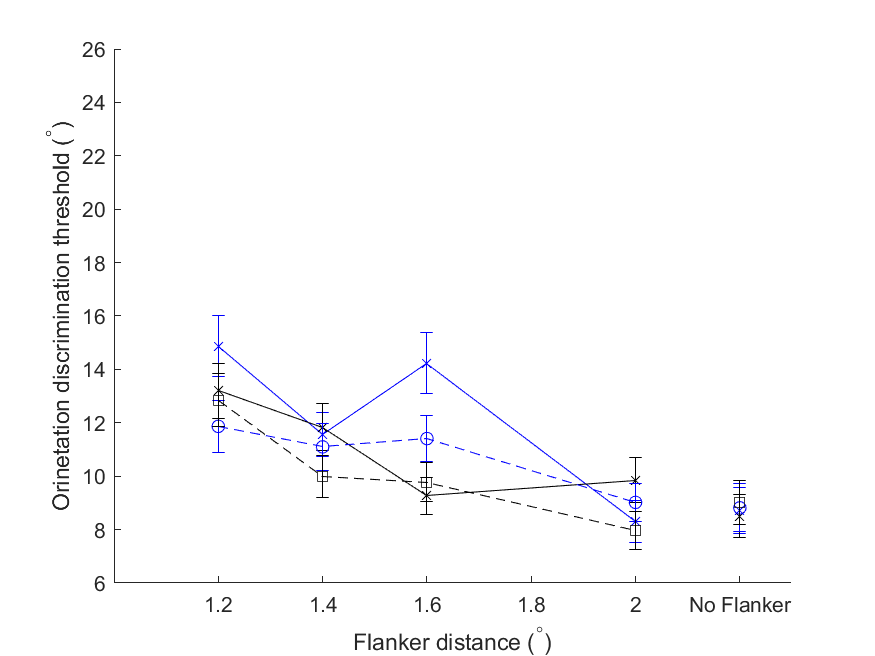


Observer: 4 (no eye tracking)


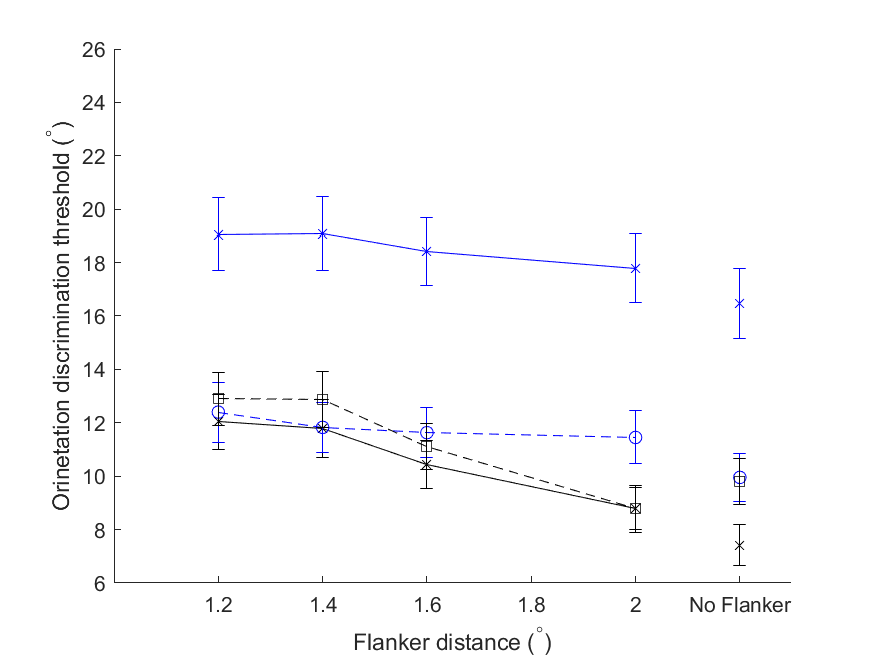


Observer: 5 (no eye tracking)


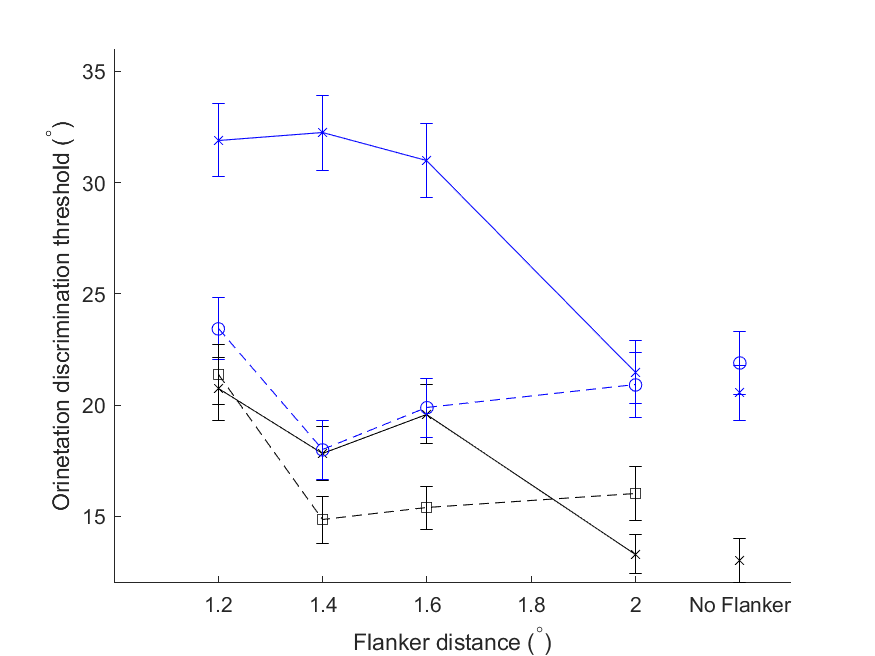


## Observers with multiplier < 1.5

Observer: 6, threshold multiplier: 1.27 (no eye tracking)


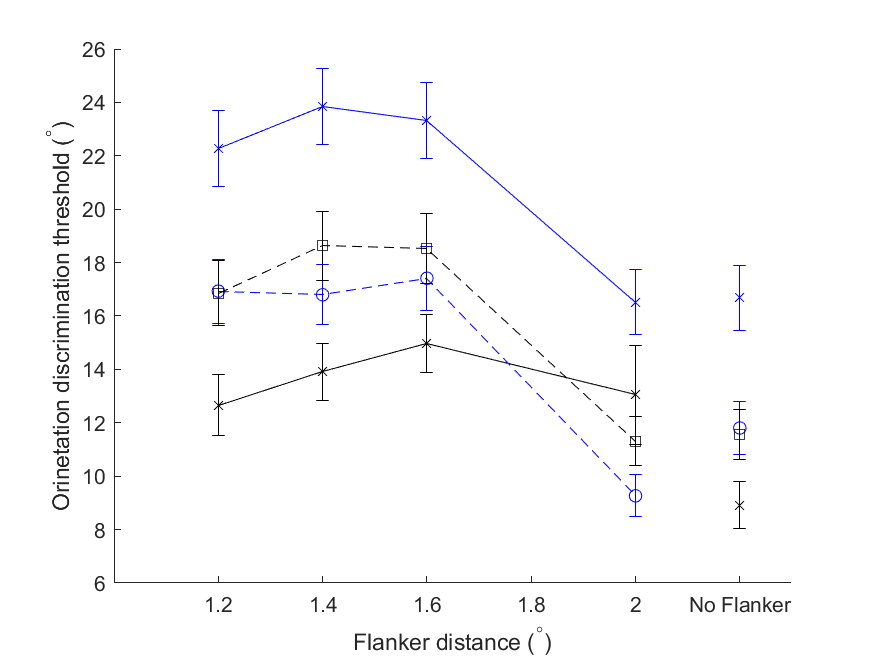


Observer: 7, threshold multiplier: 1.29 (no eye tracking)


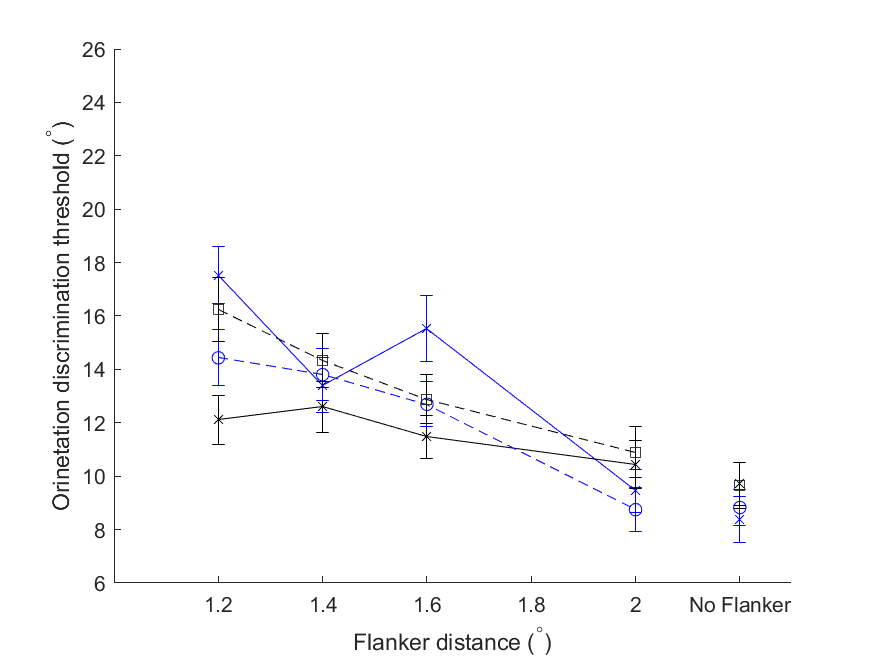


Observer: 8, threshold multiplier: 1.23 (no eye tracking)


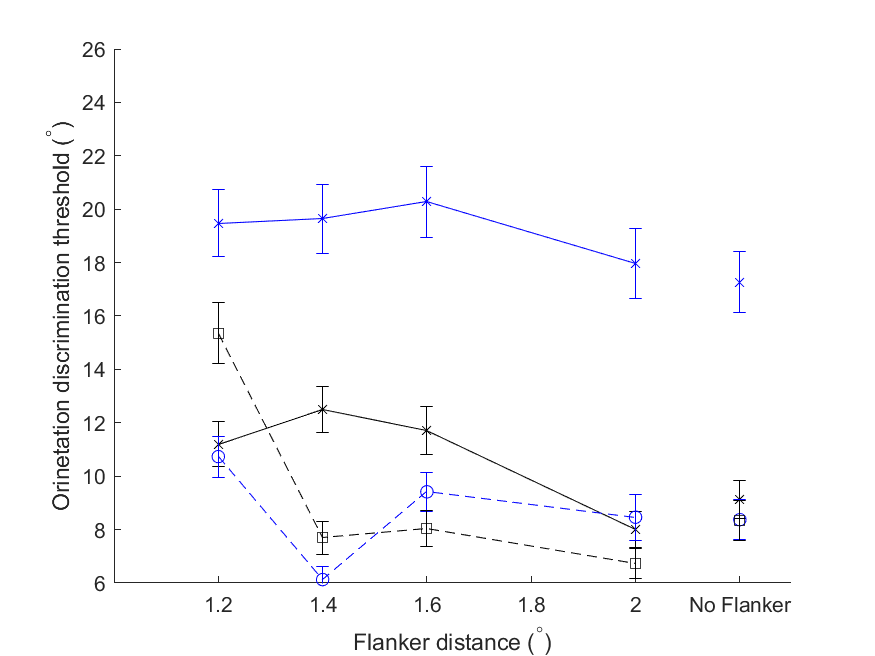


Observer 9, threshold multiplier: 1.4825 (eye tracking)
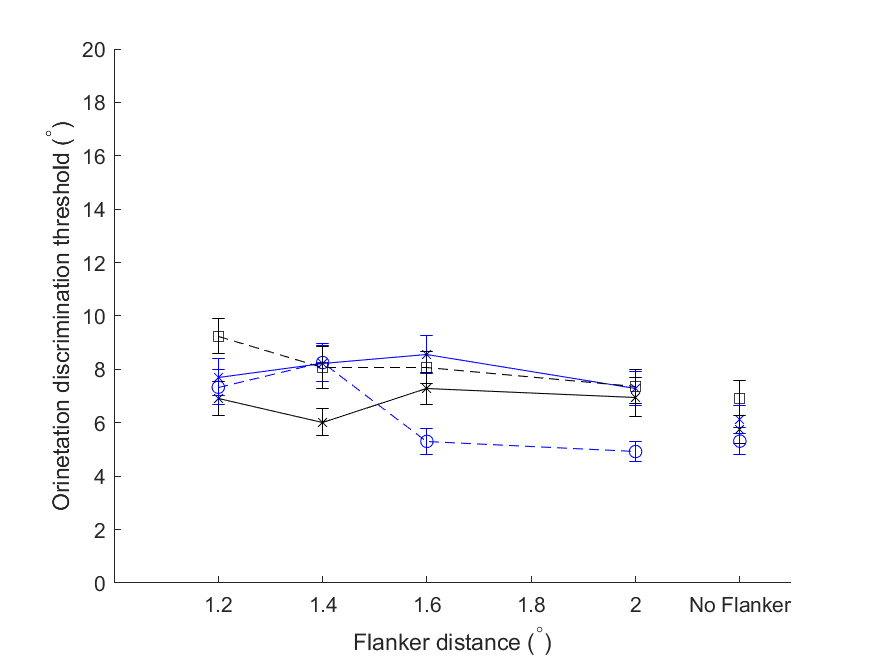


Observer 10, threshold multiplier: 1.30 (eye tracking) njjm


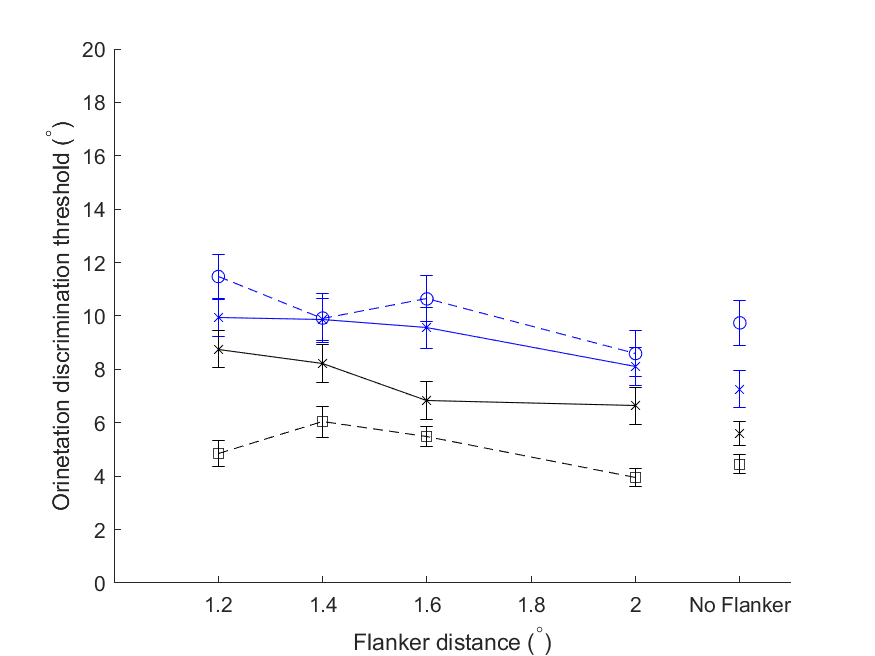


Observer 11, threshold multiplier: 1.20 (eye tracking)


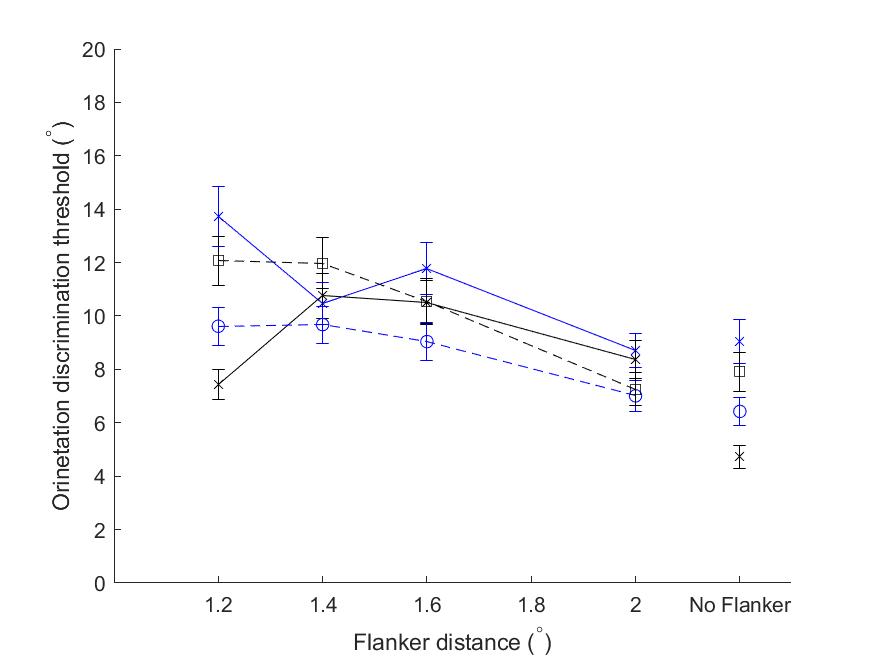

Supplement: Supplement 2 [file jovi-21-11-20_s002.docx]
